# Dementia and non-alcoholic fatty liver disease – An unprecedented relationship

**DOI:** 10.1016/j.amsu.2022.104359

**Published:** 2022-08-22

**Authors:** Fakhar Latif, Vania Saqib, Eisha Waqar

**Affiliations:** Department of Internal MCedicine, Dow University of Health Sciences, Karachi, Pakistan

Dementia, classically defined as a syndrome, is characterised by the loss of an individual's cognitive functioning due to progressive or chronic neurological deterioration. It has affected more than 50 million people worldwide, increasing to 10 million cases annually. The onset of dementia occurs most often after age 65, and women are more susceptible to the disease than men [1].

Dementia is divided into a broad range of subtypes: vascular, Alzheimer's, frontotemporal and Lewy-body. Despite their different pathophysiological mechanisms, all these subtypes share common risk factors, including obesity, inflammation, insulin resistance and blood-brain barrier abnormalities [2]. In addition, many recent studies have also proposed NAFLD as a plausible risk factor. A recent article by Shang et al. revealed a strong relationship between dementia and non-alcoholic liver disease (NAFLD) among a cohort of elderly subjects [3]. The study analysed 2898 NAFLD patients with a group of 28,357 healthy individuals and investigated the prevalence of dementia using adjusted hazard ratios (aHR) in each group. The researchers established that NAFLD patients were more likely to be predisposed to dementia (aHR = 1.38) or vascular dementia (aHR = 1.44) than healthy subjects. Furthermore, the study also observed that dementia increases twofold among patients suffering from cardiovascular diseases (CVDs) and NAFLD. These results align with another research, which exhibited a similar correlation between NAFLD and dementia [4]. This study employed Mini-Mental State Examination (MMSE) as the primary parameter to evaluate cognitive functioning in NAFLD patients, where MMSE is inversely related to cognitive function. It was observed that NAFLD patients had notably lower mean values of MMSE, concluding that NAFLD can potentially elevate the risk of contracting dementia. In another study, Seo et al. discovered that patients with NAFLD suffered from a memory deficit and a low attention span. Other symptoms of dementia such as partial loss of visuospatial function and poor thinking speed were also observed [5].

There is strong evidence available which may explain how NAFLD can cause dementia. One such example is neurotoxicity caused by hyperammonemia, which occurs in NAFLD due to the inhibition of the urea cycle [6]. This modifies the permeability of the blood-brain barrier and affects neurotransmission across the central nervous system [7]. Apart from this, NAFLD can lead to marked inflammation via cytokine storm and activation of proteolytic enzymes, such as 5-Lipoxygenase (5-LO) [8]. This may result in neurodegeneration of the hippocampus, a structure that primarily deals with long-term memory [9]. NAFLD also shows disruption of gut microbiota with low bacterial diversity and greater Firmicutes/Bacteroidetes [10]. These factors are known to increase gut permeability and insulin resistance, contributing to the development of steatosis in the liver. When gut dysbiosis occurs, it disrupts the tight junction barrier and causes the release of various pathogenic and toxic substances that can inhibit anti-inflammatory mechanisms [2]. This can enable excessive secretion of pro-inflammatory mediators that lead to eventual inflammation of cerebral tissue. Hence, this is likely how NAFLD-related gut dysbiosis, hyperammonemia and inflammation can result in the advent of dementia among the affected individuals.

The cohort study also revealed that NAFLD could potentially lead to the development of vascular dementia, but not Alzheimer's disease (AD). This contrasts another study conducted on a larger scale and demonstrated a positive association between NAFLD and AD. They used fatty liver index (FLI) to define NAFLD and discovered that a high FLI is linked with an elevated risk of AD (7.7%) [11]. Apart from this, Shang et al. also did not include Lewy Body Dementia (LBD) which is the second most common subtype after AD [12]. This is important because LBD and AD share similar pathophysiology and symptoms [13]. Hence, future large-scale trials are required to find a connection between NAFLD with all types of dementia. Furthermore, Shang et al. used the National Patients Registry (NPR) to identify patients diagnosed with NAFLD. This means that the research was only conducted on patients in secondary or tertiary care who were suffering from a more severe form of NAFLD. Therefore, it is still unknown if the early stages of NAFLD can escalate the risk of dementia.

Nevertheless, the study provided potential evidence of dementia as a likely complication in NAFLD, which is already becoming increasingly prevalent worldwide. Thus, taking measures to control NAFLD in vulnerable patients is paramount to reducing the incidence of cognitive impairment, eventually leading to dementia. It is important to note that NAFLD is usually asymptomatic and is often diagnosed after the advancement of the disease [14]. A golden rule, in this case, could be an early screening of patients with risk factors, such as obesity, diabetes type II, and dyslipidaemia. Abdominal ultrasounds can be used for diagnosis, and MRIs can be used to distinguish NAFLD from NASH [15]. People must also be counselled to avoid diets high in triglycerides and to choose an active lifestyle, so NAFLD can be avoided and hence the risk of dementia is reduced [16]. Several studies have also proposed pharmacotherapy for NAFLD through SGLT2 inhibitors, statins and Sirtuin-1, 3 and 4 to reduce inflammation, insulin resistance, and oxidative stress [17]. As these factors can potentially cause dementia in NAFLD patients, these drugs can prevent the advancement of both diseases.

In conclusion, abundant evidence suggests that NAFLD and dementia are interlinked. It has become more than essential to work on the possible therapies for NAFLD, given that it results in severe complications. Although the study by Shang et al. had its limitations, it exposed the potential causation between NAFLD and dementia, giving a new dimension to the study of these diseases.

## Ethical approval

Not Applicable.

## Sources of funding

The authors have no sources of funding to declare.

## Author contribution

Fakhar Latif – study concept or design, writing the paper. Vania Saqib – study concept or design, writing the paper. Eisha Waqar – study concept or design, contributor (reviewing).

## Research registration number

1. Name of the registry:

2. Unique Identifying number or registration ID:

3. Hyperlink to your specific registration (must be publicly accessible and will be checked):

## Guarantor

Eisha Waqar.

## Consent

Not Applicable.

## Declaration of competing interest

The authors declare that they have no competing interest.
